# The correlation between admission hyperglycemia and 30-day readmission after hip fracture surgery in geriatric patients: a propensity score-matched study

**DOI:** 10.3389/fendo.2024.1340435

**Published:** 2024-02-21

**Authors:** Wanyun Tang, Xiaomin Ni, Wei Yao, Wei Wang, Qiaomei Lv, Wenbo Ding, Renjian He

**Affiliations:** ^1^ Department of Orthopedics, Zigong First People’s Hospital, Zigong, China; ^2^ Department of Orthopedics, Dandong Central Hospital, China Medical University, Dandong, China; ^3^ Department of Orthopedics, Zigong Fourth People’s Hospital, Zigong, China; ^4^ Department of Endocrinology, Dandong Central Hospital, China Medical University, Dandong, China

**Keywords:** hip fracture, 30-day readmission, hyperglycemia, risk factor, geriatric

## Abstract

**Purpose:**

This study aimed to investigate the association between admission hyperglycemia and 30-day readmission after hip fracture surgery in geriatric patients.

**Methods:**

This retrospective study included 1253 geriatric hip fracture patients. Patients were categorized into normoglycemia(<6.10 mmol/L) and hyperglycemia groups(≥6.10 mmol/L) based on admission blood glucose. We performed multivariable logistic regression analyses and propensity score matching (PSM) to estimate adjusted odds ratios and 95% confidence intervals for 30-day readmission, controlling for potential confounding factors. An analysis of the dose-dependent association between admission blood glucose and the probability of 30-day readmission was performed. Additional subgroup analysis was conducted to examine the impact of other factors on the relationship between admission blood glucose and 30-day readmission.

**Results:**

Patients with hyperglycemia had higher 30-day readmission rates than normoglycemic patients before (19.1% vs 9.7%, p<0.001) and after PSM (18.1% vs 12.3%, p=0.035). Admission hyperglycemia was an independent predictor of increased 30-day readmission risk, with an adjusted odds ratio of 1.57 (95% CI 1.08-2.29, p=0.019) after multivariable regression and 1.57 (95% CI 1.03-2.39, p=0.036) after PSM. A dose-response relationship was observed between higher glucose levels and increased readmission risk.

**Conclusion:**

Admission hyperglycemia is an independent risk factor for 30-day readmission after hip fracture surgery in the elderly. Routine glucose testing upon admission and perioperative glycemic control may help reduce short-term readmissions in this vulnerable population.

## Introduction

Hip fractures in the elderly present a major global health concern (1). With population aging, the incidence is projected to surpass 4.5 million cases per year by 2050 (2). These fractures often occur due to falls and osteoporosis, frequently requiring surgical treatment to enable earlier rehabilitation and improved outcomes compared to conservative management (3, 4). However, surgery also carries risks of complications like infections, deep vein thrombosis, pulmonary embolism, and readmission (5, 6).

30-day readmission rates within 30 days of discharge following hip fracture surgery range from 4% to 30% (7, 8). Readmissions lead to more severe complications, extended recovery times, increased costs, and poorer outcomes (7–9). Current research has mainly focused on the effects of complications, malnutrition and frailty status on readmissions, with less attention to the influence of metabolic disorders on early readmission after hip fracture surgery in the elderly (8, 9).

Admission hyperglycemia, defined as blood glucose ≥6.1 mmol/L upon hospital admission, is a common metabolic disorder among geriatric hip fracture patients (10, 11). In most cases, admission hyperglycemia reflects stress-induced hyperglycemia resulting from the physiological trauma response (12). Specifically, the stress of the fracture initiates hormonal changes, including elevated cortisol and catecholamines, which lead to increased hepatic glucose production and reduced insulin sensitivity, contributing to high blood glucose levels (13, 14). Prolonged stress-induced hyperglycemia can impair immune function, delay wound healing, and increase infection risk (15, 16). These factors may coexist, negatively impacting 30-day readmission rates after hip fracture surgery in geriatric patients.

However, the association between admission hyperglycemia and 30-day readmission after hip fracture surgery remains unclear. Elucidating this relationship is crucial to identifying modifiable factors to reduce readmissions. Therefore, this study aimed to investigate the correlation between admission hyperglycemia and 30-day readmission after hip fracture surgery in geriatric patients using propensity score matching analysis. The Findings from this study may provide evidence to recommend routine blood glucose testing upon admission and perioperative glycemic control to improve outcomes in this vulnerable population.

## Methods

### Data sources and patient

This retrospective analysis utilizing anonymized clinical information was approved by the Institutional Review Board (IRB) at a level I trauma center. Due to the observational nature of the study and the use of de-identified patient data, the requirement for informed consent was exempted by the IRB. Data extracted from electronic medical records was limited to anonymous information of patients who underwent hip fracture surgery between November 2011 and October 2023 at our Hospital. Continuous electronic health records were retrospectively accessed for this study only to analyze anonymized data, ensuring personal health information remained protected. Data was retrospectively collected from the hospital information system on demographics, comorbidities, surgical factors, laboratory results, and complications for this study.

The study population consisted of geriatric patients who had a hip fracture diagnosis confirmed by X-ray or CT imaging and subsequently confirmed surgically. Patients were excluded from the study if they met any of the following criteria:(1) No surgical intervention; (2) Age < 60 years; (3) Pathological, old, multiple or open fractures; (4) Severe infections or malignancies; (5) Severe cardiac, hepatic or renal dysfunction, and (6) Incomplete data.

### Exposure and outcome

The primary exposure examined was hyperglycemia at hospital admission, defined as a blood glucose level of ≥6.1 mmol/L. Blood samples were obtained from hip fracture patients within 24 hours of admission to test for baseline hyperglycemia. Normal blood glucose was defined as 4.0-6.1 mmol/L. To assess the dose-response relationship between admission blood glucose and 30-day readmission after hip fracture surgery in geriatric patients, admission blood glucose levels were categorized into four quartile groups: Q1 (<5.4 mmol/L), Q2 (5.4-6.1 mmol/L), Q3 (6.1-7.5 mmol/L), and Q4 (≥7.5 mmol/L).

The main outcome evaluated was unplanned rehospitalization within 30 days of discharge after the initial hip fracture surgery. Any subsequent inpatient admission to an acute care hospital occurring within 30 days of the discharge date from the index hospitalization for hip fracture surgery was considered a 30-day readmission. The discharge date was designated as day 0. Electronic medical records were retrospectively reviewed to identify readmissions within the 30-day post-discharge. Readmission within 30 days was documented as a binary variable of yes or no for data analysis.

### Statistical analysis

For descriptive analysis, categorical factors were summarized using percentages (%) and group comparisons made using chi-square tests. Continuous measures were reported as mean ± standard deviation (SD) and analyzed between the two groups with independent sample t-tests. One-way ANOVA was used to compare the mean values of continuous variables among multiple groups.

The association between blood glucose levels and 30-day hospital readmission was examined using logistic regression models. Potential confounding factors with p-values ≥0.05 were adjusted for in univariate logistic regression analyses, while variables with p-values <0.05 were included in subsequent multivariate logistic regression models. The area under the ROC curve (AUC) was calculated to determine the ability of blood glucose levels to differentiate between 30-day readmission and non-30-day readmission patients. In addition, a restricted cubic spline plot was generated to flexibly model the association between continuous glucose levels and 30-day readmission risk using 3 knots. The association between glucose levels and the risk of 30-day readmission was assessed by plot of observed rates and predicted probabilities against glucose levels.

To minimize the potential confounding effects of covariates, we employed propensity score matching (PSM) using the nearest neighbor algorithm in a 1:1 ratio to ensure balanced covariates between the groups. A caliper width of 0.25 standard deviations (SD) was applied to match group characteristics, assessed by standardized mean differences (SMDs). Subsequently, a subgroup analysis was conducted within the PSM cohort to investigate the diagnostic utility of admission blood glucose. Within the PSM cohort, stratification was performed based on all covariates, and univariate logistic regression analysis was conducted to examine the association between hyperglycemia and 30-day readmission. Odds ratios (ORs) and corresponding 95% confidence intervals (CIs) were calculated to quantify the strength of the association.

All statistical analyses were performed using SPSS version 26 (IBM Corp., Armonk, NY, USA) and R software version 4.0.3 (R Foundation for Statistical Computing, Vienna, Austria).

## Results

### Baseline characteristics

According to exclusion criteria, 1021 patients were excluded. 1253 patients were included in the final statistical analysis. (**eFigure1**). Patients in the readmission group exhibited a higher mean age compared to those without readmission. Additionally, they had a higher prevalence of comorbidities and complications. (**Supplementary eTable 1**). **Table 1** shows the baseline characteristics of patients stratified into quartiles by admission blood glucose level. Higher glucose quartiles had older patients, more comorbidities, higher ASA scores, and more postoperative complications like deep vein thrombosis and urinary tract infection.

**Table 1:** Baseline characteristics of the patients by admission blood glucose levels(mmol/L) (Quartile-based four-category).

Variables	Total patients (n = 1253)	Admission blood glucose levels (mmol/L)				^†^ p values
		Q1 (< 5.4) (n = 306)	Q2 ((5.4-6.1) (n=283)	Q3 (6.1-7.5) (n=347)	Q4 (≥7.5) (n =317)	
Demographic						
Male gender (n, %)	499 (39.8)	138 (45.1)	124 (43.8)	140 (40.3)	97 (30.6)	0.001
Age, × year (Mean, SD)	74.72 (9.58)	71.85 (9.92)	73.41 (9.61)	76.05 (9.25)	77.21 (8.67)	<0.001
Smoking (n, %)	215 (17.2)	61 (19.9)	52 (18.4)	56 (16.1)	46 (14.5)	0.287
Alcohol (n, %)	146 (11.7)	35 (11.4)	40 (14.1)	43 (12.4)	28 (8.8)	0.227
Comorbidities						
Hypertension (n, %)	632 (50.4)	112 (36.6)	124 (43.8)	185 (53.3)	211 (66.6)	<0.001
Diabetes (n, %)	286 (22.8)	20 (6.5)	24 (8.5)	50 (14.4)	192 (60.6)	<0.001
COPD (n, %)	146 (11.7)	30 (9.8)	24 (8.5)	43 (12.4)	49 (15.5)	0.038
Cardiovascular disease (n, %)	384 (30.6)	74 (24.2)	80 (28.3)	97 (28.0)	133 (42.0)	<0.001
Stroke (n, %)	328 (26.2)	61 (19.9)	68 (24.0)	92 (26.5)	107 (33.8)	0.001
Dementia(n, %)	48 (3.8)	12 (3.9)	8 (2.8)	18 (5.2)	10 (3.2)	0.406
Intracerebral hemorrhage (n, %)	67 (5.3)	13 (4.2)	9 (3.2)	20 (5.8)	25 (7.9)	0.057
Chronic liver disease (n, %)	58 (4.6)	12 (3.9)	11 (3.9)	12 (3.5)	23 (7.3)	0.081
Chronic kidney disease (n, %)	64 (5.1)	12 (3.9)	16 (5.7)	20 (5.8)	16 (5.0)	0.712
Operation						
Fracture type						
Femoral neck fracture (n, %)	663 (52.9)	204 (66.7)	154 (54.4)	161 (46.4)	144 (45.4)	<0.001
Intertrochanteric fracture (n, %)	516 (41.2)	83 (27.1)	116 (41.0)	167 (48.1)	150 (47.3)	
Subtrochanteric fracture (n, %)	74 (5.9)	19 (6.2)	13 (4.6)	19 (5.5)	23 (7.3)	
Surgery type						
Total Hip Arthroplasty (n, %)	160 (12.8)	56 (18.3)	34 (12.0)	27 (7.8)	43 (13.6)	<0.001
Hemiarthroplasty (n, %)	309 (24.7)	68 (22.2)	68 (24.0)	92 (26.5)	81 (25.6)	
Intramedullary nail fixation (n, %)	414 (33.0)	67 (21.9)	91 (32.2)	129 (37.2)	127 (40.1)	
Internal fixation with steel plate (n, %)	166 (13.2)	29 (9.5)	35 (12.4)	57 (16.4)	45 (14.2)	
Internal fixation with hollow nails (n, %)	204 (16.3)	86 (28.1)	55 (19.4)	42 (12.1)	21 (6.6)	
Intraoperative blood loss, ×ml (Mean, SD)	175.20 (153.37)	159.12 (154.56)	167.24 (153.36)	182.43 (139.40)	189.91 (165.21)	0.041
Transfusion (n, %)	208 (16.6)	32 (10.5)	47 (16.6)	66 (19.0)	63 (19.9)	0.007
Postoperative ICU (n, %)	59 (4.7)	9 (2.9)	10 (3.5)	20 (5.8)	20 (6.3)	0.129
Admission time						
>6 hours (n, %)	666 (53.2)	136 (44.4)	153 (54.1)	205 (59.1)	172 (54.3)	0.002
6-24 hours (n, %)	192 (15.3)	47 (15.4)	47 (16.6)	56 (16.1)	42 (13.2)	
≥ 24 hours (n, %)	395 (31.5)	123 (40.2)	83 (29.3)	86 (24.8)	103 (32.5)	
Bedridden time, ×day (Mean, SD)	5.90 (4.02)	5.14 (3.06)	5.61 (3.55)	6.00 (4.64)	6.76 (4.35)	0.400
Intraoperative time, ×hour (Mean, SD)	1.66 (0.80)	1.59 (0.78)	1.61 (0.71)	1.72 (0.82)	1.72 (0.88)	0.999
ASA classification						
III-IV (n, %)	705 (56.3)	146 (47.7)	145 (51.2)	196 (56.5)	218 (68.8)	<0.001
I-II (n, %)	548 (43.7)	160 (52.3)	138 (48.8)	151 (43.5)	99 (31.2)	
Laboratory findings						
WBC count, ×10^9/L (Mean, SD)	8.86 (2.86)	8.03 (2.54)	8.58 (2.71)	9.19 (3.03)	9.54 (2.89)	0.656
HGB level, ×g/L (Mean, SD)	119.72 (20.58)	121.99 (19.85)	120.90 (21.51)	118.61 (20.08)	117.67 (20.77)	0.008
Albumin , ×gl/L (Mean, SD)	37.95 (4.62)	38.52 (4.74)	38.29 (4.46)	37.65 (4.61)	37.43 (4.58)	0.169
BUN,×mmol/L (Mean, SD)	7.40 (4.87)	6.98 (4.06)	6.87 (5.67)	7.53 (4.73)	8.14 (4.90)	0.059
Cr, ×umol/L (Mean, SD)	72.37 (64.79)	74.39 (78.63)	67.67 (43.69)	67.98 (33.64)	79.40 (87.34)	0.228
D-Dimer, ×mg/L (Mean, SD)	4.94 (5.04)	4.00 (4.33)	4.65 (4.63)	5.44 (5.34)	5.58 (5.55)	0.005
Common complication						
DVT (n, %)	160 (12.8)	18 (5.9)	22 (7.8)	47 (13.5)	73 (23.0)	<0.001
UTI (n, %)	289 (23.1)	26 (8.5)	44 (15.5)	88 (25.4)	131 (41.3)	<0.001
Pneumonia (n, %)	114 (9.1)	6 (2.0)	10 (3.5)	48 (13.8)	50 (15.8)	<0.001

COPD, Chronic obstructive pulmonary disease; ASA, the American Society of Anesthesiologists Physical Status Classification System; WBC, White blood cell; HGB, hemoglobin; BUN, Blood urea nitrogen; Cr, Creatinine; DVT, Deep Vein Thrombosis; UTI, Urinary Tract Infection. ^†^ p values for continuous variables are from An analysis of variance (ANOVA), and categorical variables are from chi-square tests.

### Multivariate analysis

To explore the association between 32 factors and 30-day readmission, both univariate and multivariate analyses were conducted (**Table 2**). After controlling for potential confounding variables, four factors emerged as independent predictors of 30-day readmission: age, intracerebral hemorrhage, ASA classification, and blood glucose levels. These factors retained their significance even after adjusting for other variables.

**Table 2:** Univariate and multivariate analysis for 30-day readmission after hip fracture surgery in geriatric patients.

Variables	Univariate			Multivariate		
	OR	95%CI	p-value	OR	95%CI	p-value
Demographic						
Male gender	0.76	0.55-1.05	0.095	<NA>	<NA>	<NA>
Age	1.06	1.04-1.08	<0.001	1.03	1.01-1.06	0.002
Smoking	0.85	0.55-1.30	0.450	<NA>	<NA>	<NA>
Alcohol	0.74	0.44-1.26	0.271	<NA>	<NA>	<NA>
Comorbidities						
Hypertension	1.88	1.36-2.60	<0.001	1.27	0.89-1.80	0.185
Diabetes	1.60	1.13-2.26	0.008	1.08	0.70-1.68	0.724
COPD	1.45	0.93-2.26	0.104	<NA>	<NA>	<NA>
Cardiovascular disease	1.40	1.01-1.94	0.045	0.82	0.57-1.18	0.283
Stroke	1.93	1.39-2.68	<0.001	1.33	0.94-1.89	0.113
Dementia	1.77	0.89-3.54	0.105	<NA>	<NA>	<NA>
Intracerebral hemorrhage	2.26	1.28-3.97	0.005	2.013	1.17-3.87	0.013
Chronic liver disease	1.38	0.70-2.72	0.348	<NA>	<NA>	<NA>
Chronic kidney disease	2.03	1.124-3.65	0.019	1.58	0.86-2.92	0.143
Operation						
Fracture type	1.12	0.86-1.46	0.392	<NA>	<NA>	<NA>
Surgery type	0.81	0.71-0.92	0.001	0.87	0.74-1.02	0.087
Intraoperative blood loss,	1.00	1.00-1.00	0.379	<NA>	<NA>	<NA>
Transfusion	1.32	0.89-1.96	0.167	<NA>	<NA>	<NA>
Postoperative ICU	1.05	0.51-2.17	0.899	<NA>	<NA>	<NA>
Admission time	1.16	0.98-1.38	0.094	<NA>	<NA>	<NA>
Bedridden time	1.01	0.98-1.05	0.437	<NA>	<NA>	<NA>
Intraoperative time	0.83	0.66-1.03	0.091	<NA>	<NA>	<NA>
ASA classification	3.15	2.18-4.54	<0.001	1.92	1.29-2.86	0.001
Laboratory findings						
WBC count	1.05	0.99-1.10	0.083	<NA>	<NA>	<NA>
HGB level	0.99	0.98-0.99	0.001	1.00	0.99-1.01	0.622
Albumin	0.93	0.90-0.96	<0.001	0.97	0.93-1.01	0.114
BUN	1.02	0.99-1.05	0.166	<NA>	<NA>	<NA>
Cr	1.00	1.00-1.00	0.765	<NA>	<NA>	<NA>
D-Dimer	1.02	0.99-1.05	0.281	<NA>	<NA>	<NA>
Blood glucose	1.12	1.06-1.17	<0.001	1.07	1.01-1.15	0.040
Common complication						
DVT	1.70	1.12-2.57	0.013	1.08	0.68-1.71	0.749
UTI	1.38	0.97-1.97	0.071	<NA>	<NA>	<NA>
Pneumonia	2.53	1.63-3.95	<0.001	1.55	0.96-2.49	0.073

p-value is from Univariate and Multivariate (P<0.05 indicates statistical significance).

COPD, Chronic obstructive pulmonary disease; ASA, the American Society of Anesthesiologists Physical Status Classification System; WBC, White blood cell; HGB, hemoglobin; BUN, Blood urea nitrogen; Cr, Creatinine; DVT, Deep Vein Thrombosis; UTI, Urinary Tract Infection.

### Propensity score matching

The baseline characteristics of patients stratified into <6.1 mmol/L and ≥6.1 mmol/L groups before and after 1:1 propensity score matching (PSM) are presented in **Table 3**. Propensity score matching achieved a good balance between glucose <6.1 mmol/L and ≥6.1 mmol/L groups, with standardized mean differences <0.1 for most variables.

**Table 3:** Patient characteristics before and after propensity score matching by admission blood glucose (≥ 6.10 mmol/L and < 6.10 mmol/L).

Variables	Before PSM (N=1253)			After PSM (N=698)		
	Glucose < 6.10 (n=589)	Glucose ≥ 6.10 (n=664)	SMD	Glucose < 6.10 (n=349)	Glucose ≥ 6.10 (n=349)	SMD
Demographic						
Male gender (n, %)	262 (44.5)	237 (35.7)	0.426	143 (41.0)	141 (40.4)	0.041
Age, × year (Mean, SD)	72.60 (9.79)	76.61 (8.99)	0.180	75.55 (9.74)	75.16 (9.47)	0.012
Smoking (n, %)	113 (19.2)	102 (15.4)	0.101	61 (17.5)	60 (17.2)	0.008
Alcohol (n, %)	75 (12.7)	71 (10.7)	0.063	42 (12.0)	44 (12.6)	0.017
Comorbidities						
Hypertension (n, %)	236 (40.1)	396 (59.6)	0.399	179 (51.3)	187 (53.6)	0.046
Diabetes (n, %)	44 (7.5)	242 (36.4)	0.747	44 (14.6)	50 (14.3)	0.050
COPD (n, %)	54 (9.2)	92 (13.9)	0.147	30 (8.6)	34 (9.7)	0.040
Cardiovascular disease (n, %)	154 (26.1)	230 (34.6)	0.185	111 (31.8)	110 (31.5)	0.006
Stroke (n, %)	129 (21.9)	199 (30.)	0.185	94 (26.9)	94 (26.9)	<0.001
Dementia(n, %)	20 (3.4)	28 (4.2)	0.043	15 (4.3)	15 (4.3)	<0.001
Intracerebral hemorrhage (n, %)	22 (3.7)	45 (6.8)	0.137	17 (4.9)	19 (5.4)	0.026
Chronic liver disease (n, %)	23 (3.9)	35 (5.3)	0.065	12 (3.4)	21 (6.0)	0.122
Chronic kidney disease (n, %)	28 (4.8)	36 (5.4)	0.030	16 (4.6)	22 (6.3)	0.076
Operation						
Fracture type						
Femoral neck fracture (n, %)	358 (60.8)	305 (45.9)	0.262	182 (52.1)	175 (50.1)	0.028
Intertrochanteric fracture (n, %)	199 (33.8)	317 (47.7)		143 (41.0)	151 (43.3)	
Subtrochanteric fracture (n, %)	32 (5.4)	42 (6.3)		24 (6.9)		
Surgery type						
Total Hip Arthroplasty (n, %)	90 (15.3)	70 (10.5)	0.144	45 (12.9)	35 (10.0)	0.024
Hemiarthroplasty (n, %)	136 (23.1)	173 (26.1)		88 (25.2)	99 (28.4)	
Intramedullary nail fixation (n, %)	158 (26.8)	256 (38.6)		114 (32.7)	115 (33.0)	
Internal fixation with steel plate (n, %)	64 (10.9)	102 (15.4)		44.0 (12.6)	59 (16.9)	
Internal fixation with hollow nails (n, %)	141 (23.9)	63 (9.5)		58 (16.6)	41 (11.7)	
Intraoperative blood loss, ×ml (Mean, SD)	196.56 (158.95)	169.94 (150.94)	0.150	176.05 (170.26)	177..97 (137.27)	0.012
Transfusion (n, %)	79 (13.4)	129 (19.4)	0.163	62 (17.8)	67 (19.2)	0.037
Postoperative ICU (n, %)	19 (3.2)	40 (6.0)	0.133	15 (4.3)	12 (3.4)	0.045
Admission time						
>6 hours (n, %)	289 (49.1)	377 (56.8)	0.159	193 (55.3)	196 (56.2)	0.010
6-24 hours (n, %)	94 (16.0)	98 (14.8)		59 (16.9)	50 (14.3)	
≥ 24 hours (n, %)	206 (35.0)	189 (28.5)		97 (27.8)	103 (29.5)	
Bedridden time, ×day (Mean, SD)	5.37 (3.31)	6.36 (4.52)	0.251	5.84 (3.67)	5.89 (4.74)	0.014
Intraoperative time, ×hour (Mean, SD)	1.60 (0.75)	1.72 (0.85)	0.153	1.64 (0.76)	1.67 (10.76)	0.037
ASA classification						
III-IV (n, %)	291 (49.4)	414 (62.3)	0.263	197 (56.4)	201 (57.6)	0.023
I-II (n, %)	298 (50.6)	250 (37.7)		152 (43.6)	148 (42.4)	
Laboratory findings						
WBC count, ×10^9/L (Mean, SD)	8.30 (2.64)	9.36 (2.97)	0.377	8.86 (2.65)	8.86 (2.47)	0.002
HGB level, ×g/L (Mean, SD)	121.47 (20.65)	118.16 (20.40)	0.161	118.96 (21.18)	118.50 (19.54)	0.023
Albumin, ×g/L (Mean, SD)	38.41 (4.61)	37.54 (4.59)	0.188	37.75 (4.56)	37.80 (4.67)	0.012
BUN,×mmol/L (Mean, SD)	6.93 (4.90)	7.82 (4.82)	0.184	7.33 (5.75)	7.56 (3.77)	0.048
Cr, ×umol/L (Mean, SD)	71.16 (64.30)	73.43 (65.26)	0.035	67.41 (37.19)	76.58 (81.74)	0.144
D-Dimer, ×mg/L (Mean, SD)	4.31 (4.87)	5.50 (5.44)	0.239	4.85 (4.77)	4.88 (4.95)	0.006
Common complication						
DVT (n, %)	40 (6.8)	120 (18.1)	0.347	38 (10.9)	32 (9.2)	0.057
UTI (n, %)	70 (11.9)	219 (33.0)	0.522	65 (18.6)	66 (18.9)	0.007
Pneumonia (n, %)	16 (2.7)	98 (14.8)	0.436	16 (4.6)	15 (4.3)	0.014

SMD, standardized mean difference; SD, Standard deviation; PSM, propensity score matching; COPD, Chronic obstructive pulmonary disease; ASA: the American Society of Anesthesiologists Physical Status Classification System; WBC, White blood cell; HGB, hemoglobin; BUN, Blood urea nitrogen; Cr, Creatinine; DVT, Deep Vein Thrombosis; UTI, Urinary Tract Infection.

### Correlation between admission hyperglycemia and 30-day readmission

**Figure 1:**
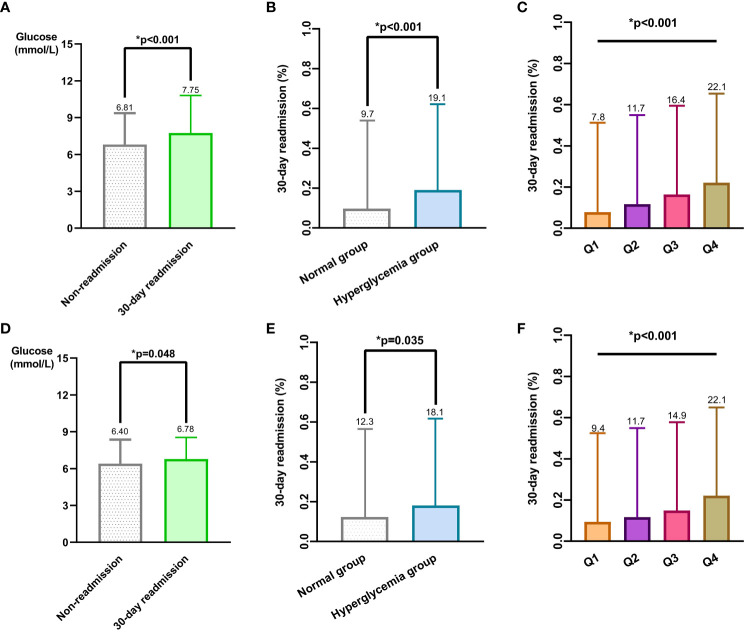
Relationship between different admission blood glucose level groups and 30-day readmission rates in patients with hip fracture before and after PSM. **(A)** Mean and standard deviation of admission blood glucose levels between the 30-day readmission group and non-readmission group before PSM. **(B)** Comparison of 30-day readmission rates between normal admission blood glucose group and hyperglycemia group before PSM. **(C)** Patients were divided into 4 quartile groups(Q1, Q2, Q3, Q4) before PSM, comparing 30-day readmission rates among the 4 quartile groups. **(D)** Mean and standard deviation of admission blood glucose levels between the 30-day readmission group and non-readmission group after PSM. **(E)** Comparison of 30-day readmission rates between normal admission blood glucose group and hyperglycemia group after PSM. **(F)** Patients were divided into 4 quartile groups after PSM, comparing 30-day readmission rates among the 4 quartile groups.

Glucose levels were significantly higher in readmission patients than non-readmission patients, both before(7.75 mmol/L vs 6.81 mmol/L, p<0.001) and after matching (6.78 mmol/L vs 6.40 mmol/L, p=0.048) (**Figures 1A, D**). Patients with hyperglycemia had higher 30-day readmission rates than normoglycemic patients before (19.1% vs 9.7%, p<0.001) and after PSM (18.1% vs 12.3%, p=0.035) (**Figures 1B, E**, **Table 4**). Patients with hyperglycemia had a higher 30-day readmission risk than normoglycemic (unadjusted OR 2.21, 95% CI 1.58-3.09, p<0.001). This remained significant after adjusting for confounders (adjusted OR 1.57, 95% CI 1.08-2.29, p=0.019). Propensity score matching to minimize selection bias yielded consistent results (PSM-adjusted OR 1.57, 95% CI 1.03-2.39, p=0.036) (**Table 5**). Moreover, analyzing glucose as a continuous variable yielded consistent results. For every 1 mmol/L increase in admission glucose, the unadjusted odds of 30-day readmission increased by 12% and the adjusted odds increased by 7%.

**Table 4:** Comparison of the incidence of 30-day readmission before and after PSM based on admission blood glucose levels (≥ 6.10 mmol/L and < 6.10 mmol/L).

Hematologic parameters	No.(%) Clinical cutoffs	Before PSM		*p-value	No.(%) Clinical cutoffs	After PSM		*p-value
		Without 30-day readmission	30-day readmission			Without 30-day readmission	30-day readmission	
Cutoff value	< 6.10 (n=589) (n=1014)	532 (90.3)	57 (9.7)	<0.001	< 6.10 (n=349) (n=1014)	306 (87.7)	43 (12.3)	0.035
	≥ 6.10 (n=664)	537 (80.9)	127 (19.1)		≥ 6.10 (n=349)	286 (81.9)	63 (18.1)	

PSM, propensity scores matching.

*p-value is from Chi-Squared Test to indicate significant differentiation (p<0.05 means significant differentiation).

**Table 5:** Unadjusted and adjusted association between admission blood glucose levels and 30-day readmission.

Type	Blood glucose level (mmol/L)	Events, n (%)	Unadjusted OR	p* trend 1	Multivariable Regression adjusted OR	p* trend 2	PSM adjusted OR	p* trend 3
Continuous	Per 1	NA	1.12 (1.06-1.17)	<0.001	1.07 (1.01-1.15)	0.040	NA	NA
Cutoff value	< 6.10	57 (9.7)	1 [Reference]	<0.001	1 [Reference]	0.019	1 [Reference]	0.036
	≥ 6.10	127 (19.1)	2.21 (1.58-3.09)		1.57 (1.08-2.29)		1.57 (1.03-2.39)	
Quartile	Q1(<5.4)	24 (7.8)	1 [Reference]	NA	1 [Reference]	NA	1 [Reference]	NA
	Q2(5.4–6.1)	33 (11.7)	1.55 (0.89-2.70)	0.120	1.57 (0.86-2.85)	0.141	1.26 (0.69-2.28)	0.070
	Q3(6.1–7.5)	57 (16.4)	2.31 (1.40-3.82)	0.001	1.73 (1.00-2.99)	0.051	1.95 (1.03-3.73)	0.042
	Q4(≥7.5)	70 (22.1)	3.33 (2.03-5.46)	<0.001	2.27 (1.22-4.21)	0.009	2.18 (1.02-4.65)	0.045

CI, confidence interval; OR, odds ratio; PSM, propensity scores matching. * P for trend.

Compared to Q1, higher glucose quartiles (Q2-Q4) had increased 30-day readmission risk after adjusting for confounders (Q2: adjusted OR 1.57, 95% CI 0.86-2.85, p=0.141; Q3: adjusted OR 1.73, 95% CI 1.00-2.99, p=0.051; Q4: adjusted OR 2.27, 95% CI 1.22-4.21, p=0.009). This association remained after propensity score matching (Q2: PSM-adjusted OR 1.26, 95% CI 0.69-2.28, p=0.070; Q3: PSM-adjusted OR 1.95, 95% CI 1.03-3.73, p=0.042; Q4: PSM-adjusted OR 2.18, 95% CI 1.02-4.65, p=0.045) (**Table 5**). In addition, the blood glucose ROC curve had an AUC of 0.625 (**eFigure 2**), indicating a moderate predictive value for 30-day readmission risk.

### Dose-response relationship

**Figure 2:**
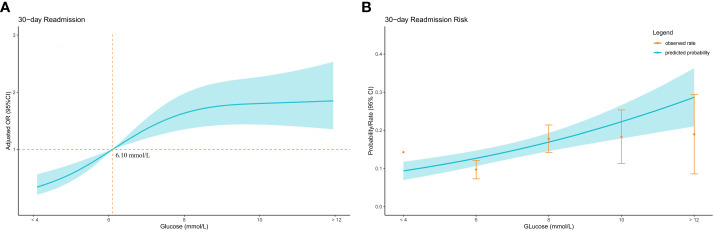
Relationship between admission blood glucose level and 30-day readmission in patients with hip fracture before PSM. **(A)** Adjusted odds ratios (ORs) and 95% confidence intervals (CIs) are shown for every 2 mmol/L deviation away from the reference value (6.1 mmol/L). **(B)** Predicted probabilities and the observed rate of 30-day readmission.

**Figure 3:**
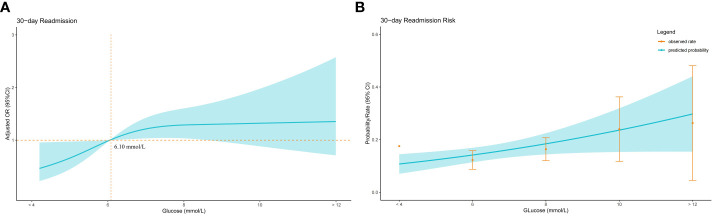
Relationship between admission blood glucose level and 30-day readmission in patients with hip fracture after PSM. **(A)** Adjusted odds ratios (ORs) and 95% confidence intervals (CIs) are shown for every 2 mmol/L deviation away from the reference value (6.1 mmol/L). **(B)** Predicted probabilities and the observed rate of 30-day readmission.

30-day readmission rates increased with increasing admission blood glucose quartile, both before(Q1 7.8%, Q2 11.7%, Q3 16.4%, Q4 22.1%) and after(Q1 9.4%, Q2 11.7%, Q3 14.9%, Q4 22.1%) PSM (**Figures 1C, F**). A dose-response relationship was observed between admission glucose and 30-day readmission before and after PSM (Before PSM: **Figure 2**; After PSM: **Figure 3**). Compared to glucose ≥6.10 mmol/L, levels <6.10 mmol/L had lower readmission risk (**Figures 2A**, **3A**). Predicted and observed readmission probabilities increased with higher admission glucose based on admission blood glucose levels, and the predicted and observed readmission probabilities are consistent (**Figures 2B**, **3B**). In summary, these data demonstrate admission hyperglycemia is an independent risk factor for 30-day readmission with a dose-response relationship.

**Figure 4:**
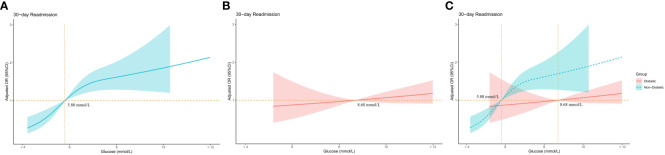
Relationship between admission blood glucose level and 30-day readmission in diabetic and non-diabetic groups. **(A)** Adjusted odds ratios (ORs) and 95% confidence intervals (CIs) are shown for every 2 mmol/L deviation away from the reference value (6.1 mmol/L) in the non-diabetic group. **(B)** Adjusted odds ratios (ORs) and 95% confidence intervals (CIs) are shown for every 2 mmol/L deviation away from the reference value (6.1 mmol/L) in the diabetic group. **(C)** Comparing adjusted odds ratios (ORs) and 95% confidence intervals (CIs) in diabetic and non-diabetic groups.

**Figure 4A** shows the adjusted odds ratios (OR) for 30-day readmission across admission blood glucose levels in non-diabetic patients. ORs became close to 1 when glucose reached 5.8 mmol/L. **Figure 4B** shows the corresponding data for diabetic patients. In contrast to non-diabetics, the OR increase is relatively slow, and it is not until the blood sugar reaches 8.10 that the OR value is greater than 1 (**Figure 4C**).

### Interaction analysis

**Figure 5:**
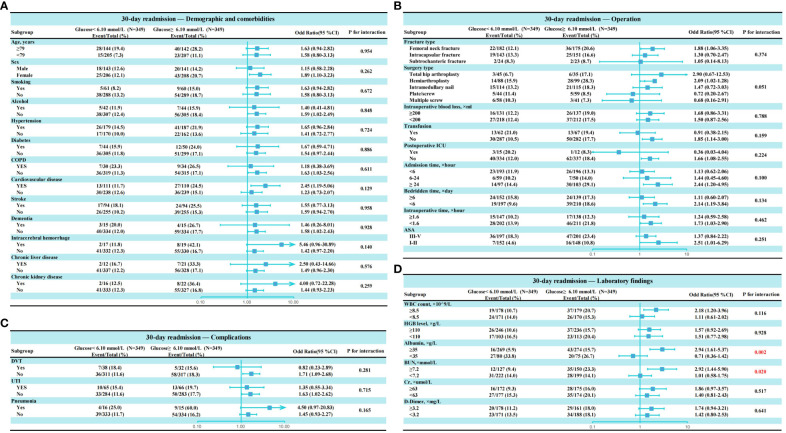
Subgroup analysis of association admission hyperglycemia and 30-day readmission after propensity score matching. **(A)** Subgroup analysis of variables related to demographics and comorbidities; **(B)** Subgroup analysis of variables related to operation. **(C)** Subgroup analysis of variables related to complications; **(D)** Subgroup analysis of variables related to laboratory findings.

This study assessed potential interactions between admission hyperglycemia and other variables (**Figures 5A-D**). We found a significant interaction between hyperglycemia and albumin levels (P=0.002), suggesting albumin status may influence the effect of hyperglycemia on 30-day readmission risk. Additionally, a significant interaction was observed between hyperglycemia and blood urea nitrogen (P=0.020), indicating blood urea nitrogen levels may also affect the impact of high blood glucose on readmission risk. This suggests albumin and blood urea nitrogen may modulate the relationship between hyperglycemia and increased 30-day readmission risk. However, no significant effect modification of the association between hyperglycemia and 30-day readmission was found for the other variables examined.

## Discussion

In this study, we found that admission hyperglycemia was independently associated with an increased risk of 30-day hospital readmission after hip fracture surgery in geriatric patients. The dose-response relationship observed between elevated glucose levels and higher readmission risk further corroborates hyperglycemia as a prognostic factor.

Notably, the association between admission hyperglycemia and readmission remained significant even after adjusting for potential demographic and clinical confounders. This indicates that hyperglycemia itself contributes to poorer short-term outcomes, rather than merely serving as a marker of greater comorbidity burden. Propensity score matching also yielded consistent results, confirming that the hyperglycemia-readmission link cannot be fully attributed to baseline differences between the groups.

Our analysis produced consistent conclusions with already published studies exploring similar hypotheses. For example, Evans et al. found that patients with high blood glucose levels (>6.5 mmol/l) upon hospital admission tended to have greater 28-day mortality rates and higher readmission frequencies in a retrospective cohort study of 1,502 patients (17). In addition, Farrugia et al. indicated admission glucose remained a significant predictor of 1-year readmission (18). Zhou et al. established the stress hyperglycemia ratio impacts the rehospitalization rate in patients with acute decompensated heart failure and diabetes (HR 1.54 [95% CI 1.03-2.32]) (19). Martin et al. conducted a study of 3,109 patients admitted to the intensive care unit and confirmed serum glucose can predict ICU readmission (20). Murphy et al. investigated 258 patients with burn injury and found patients with hyperglycemia had considerably longer hospitalizations (13 vs 9 days; P = 0.038) and greater rates of unscheduled rehospitalizations (18.8 vs 3.6%; P =0.001) compared to the control group (21).

Several mechanisms may underlie the observed relationship between hyperglycemia and increased readmission. Firstly, acute hyperglycemia can directly impair immune cell function, wound healing, and vascular epithelial integrity through advanced glycation end-products and oxidative stress (15, 16, 22–24). This may increase susceptibility to postoperative complications like surgical site infections, delirium and postoperative pneumonia, necessitating readmission. For example, Tang et al. analyzed 600 geriatric patients with hip fractures and revealed admission hyperglycemia in elderly hip fracture patients increases the risk of postoperative pneumonia more than those with normal glucose levels (OR = 2.090, 95% CI: 1.135-3.846, p = 0.016) (25). Anderson et al. demonstrated patients with blood glucose levels of 200 mg/dL or higher upon hospital admission were significantly more likely to develop deep surgical site infections in orthopaedic trauma patients (26). Windmann observed hyperglycemia was significantly associated with postoperative delirium in a prospective observational study. (OR 3.86 [CI 95% 1.13, 39.49], P=0.044) (27).

Secondly, admission hyperglycemia may be indicative of undiagnosed diabetes or poor long-term glycemic control in known diabetic patients. Sustained hyperglycemia can promote endothelial dysfunction and microvascular damage through mechanisms like advanced glycation end-product accumulation and protein kinase C activation (28–31). This macro- and microvascular dysfunction can exacerbate tissue hypoxia and ischemic injury, worsening prognosis.

Lastly, hyperglycemia induces a prothrombotic state by increasing platelet aggregation, plaque rupture risk, and impairing fibrinolysis (32, 33). Hypercoagulability coupled with endothelial injury significantly increases the risk of thromboembolic events like deep vein thrombosis and pulmonary embolism after major orthopedic surgery (34). For example, Yao et al. analyzed 217 patients with femoral neck fractures and found patients with admission hyperglycemia demonstrate a higher incidence of developing deep vein thrombosis (OR 3.03, 95% CI 0.77-11.87) (34).

Interestingly, the association between admission glucose and 30-day readmission differed between diabetics and non-diabetics in this study. The inflexion point for increased readmission odds was lower for non-diabetics compared to diabetics (5.80 mmol/L for the non-diabetic group and 8.70 mmol/L for the diabetes group). This corroborates that acute stress-induced hyperglycemia poses a greater risk in non-diabetics. In contrast, chronically elevated glucose in diabetics exerts a more gradual dose-dependent effect on outcomes. Bellis found that outcomes for patients with stress-induced hyperglycemia are worse than for patients with hyperglycemia who have diabetes in a study of acute coronary syndrome (35). Prospective studies are needed to clarify the causal relationships and mechanisms.

The interactions between admission hyperglycemia and hypoalbuminemia/renal dysfunction suggest these comorbidities exacerbate hyperglycemia’s effects. The interaction with hypoalbuminemia, indicating malnutrition, may exacerbate hyperglycemia’s deleterious effects (36, 37). Malnutrition impairs immunity and wound healing, while hyperglycemia induces glucotoxicity and oxidative stress. Their synergistic adverse effects on recovery may partly explain higher readmission rates when both factors are present. Additionally, the interaction between hyperglycemia and elevated blood urea nitrogen, indicating renal dysfunction, may amplify hyperglycemia’s impact. Nephropathy can reduce drug clearance and cause fluid/electrolyte imbalances, compounding hyperglycemia’s risks (38, 39). This interplay may increase readmission likelihood when both factors occur. In addition, relevant literature indicates that analyzing multiple subgroups may lead to false positive findings (40).

The significant interactions found between admission hyperglycemia and serum albumin as well as blood urea nitrogen warrant further discussion. Prospective studies examining their combined versus isolated impacts are needed to clarify the causal relationships and mechanisms. A better understanding of these synergistic risks will inform the management of complex patients.

Cardiovascular disease is highly prevalent in elderly hip fracture patients and an important contributor to postoperative complications and mortality. Conditions like coronary artery disease, heart failure, arrhythmias, broken heart syndrome and Takotsubo cardiomyopathy can exacerbate tissue hypoxia, limit mobility, and predispose patients to thromboembolic events, delirium and cardiac complications after major surgery (41). Acute hyperglycemia further amplifies cardiovascular risk through mechanisms like sympathoadrenal activation, oxidative stress, inflammation, and hypercoagulability (32). The synergistic adverse effects of cardiovascular disease and hyperglycemia on recovery likely contribute to higher 30-day readmission rates when both factors coexist in hip fracture patients. Managing cardiovascular comorbidities through beta blockers, statins and anticoagulation while controlling hyperglycemia may help attenuate readmissions. Future studies should examine whether optimizing cardiovascular status and glycemic control in the perioperative period improves outcomes compared to routine care.

This study has several strengths. The sample size was adequately powered to detect significant associations between hyperglycemia status and 30-day readmission. Confounding was rigorously accounted for through multivariate regression and propensity score matching. The dose-response relationship analysis visualized the relationship between admission hyperglycemia and 30-day readmission. Several limitations should also be acknowledged. The single-center retrospective design precludes establishing causality. Further prospective studies are needed to confirm these findings in other cohorts and determine causative treatments. Data was lacking on medications, surgical complications beyond 30 days, and causes for readmission. Residual confounding from unmeasured factors may still exist despite adjustment. Additionally, while this study suggests potential interventions like routine glucose testing and perioperative glycemic control, the lack of a cost-effectiveness analysis of these strategies limits the practical applicability and clinical recommendations. Future studies should investigate the cost-effectiveness of intensive perioperative glucose management protocols compared to routine care.

## Conclusions

In conclusion, this study provides evidence that admission hyperglycemia independently predicts increased 30-day readmission risk after hip fracture surgery in the elderly. Routine glucose testing upon admission and perioperative glycemic control through insulin protocols may help attenuate short-term readmissions. Our findings warrant future longitudinal studies to define optimal glycemic targets across the perioperative period. A cost-effectiveness analysis of intensive glucose management strategies is also needed. Ultimately, an evidence-based approach incorporating admission glucose status into risk stratification models could help improve the quality of care and outcomes for this vulnerable patient population.

## Data availability statement

All the data used and analyzed during the current study are available from the corresponding author upon reasonable request.

## Ethics statement

The studies involving humans were approved by the Ethics Committee of Dandong Central Hospital (No. DDZX-202211013). The studies were conducted in accordance with the local legislation and institutional requirements. Written informed consent for participation was not required from the participants or the participants’ legal guardians/next of kin in accordance with the national legislation and institutional requirements.

## Author contributions

RH: Conceptualization, Investigation, Writing – review & editing. WT: Data curation, Supervision, Writing – original draft. XN: Conceptualization, Investigation, Writing – original draft. WY: Conceptualization, Investigation, Writing – original draft. WW: Methodology, Writing – original draft. QL: Project administration, Writing – original draft. WD: Project administration, Writing – original draft.
