# Supplementary material for: The correlation between admission hyperglycemia and 30-day readmission after hip fracture surgery in geriatric patients: a propensity score-matched study
Source: Front Endocrinol (Lausanne). 2024 Feb 21;15:1340435. doi: 10.3389/fendo.2024.1340435 (PMC10915248; doi:10.3389/fendo.2024.1340435)
Supplement: Supplementary file 1 [file DataSheet_1.docx]

**Supplement**

**eFigure 1** Flow diagram of enrollment and PSM.

**eFigure 2** The prediction model of admission blood glucose levels for 30-day readmission rate after hip fracture.

**eTable 1** Baseline characteristics of the patients between 30-day readmission and non-readmission groups

**
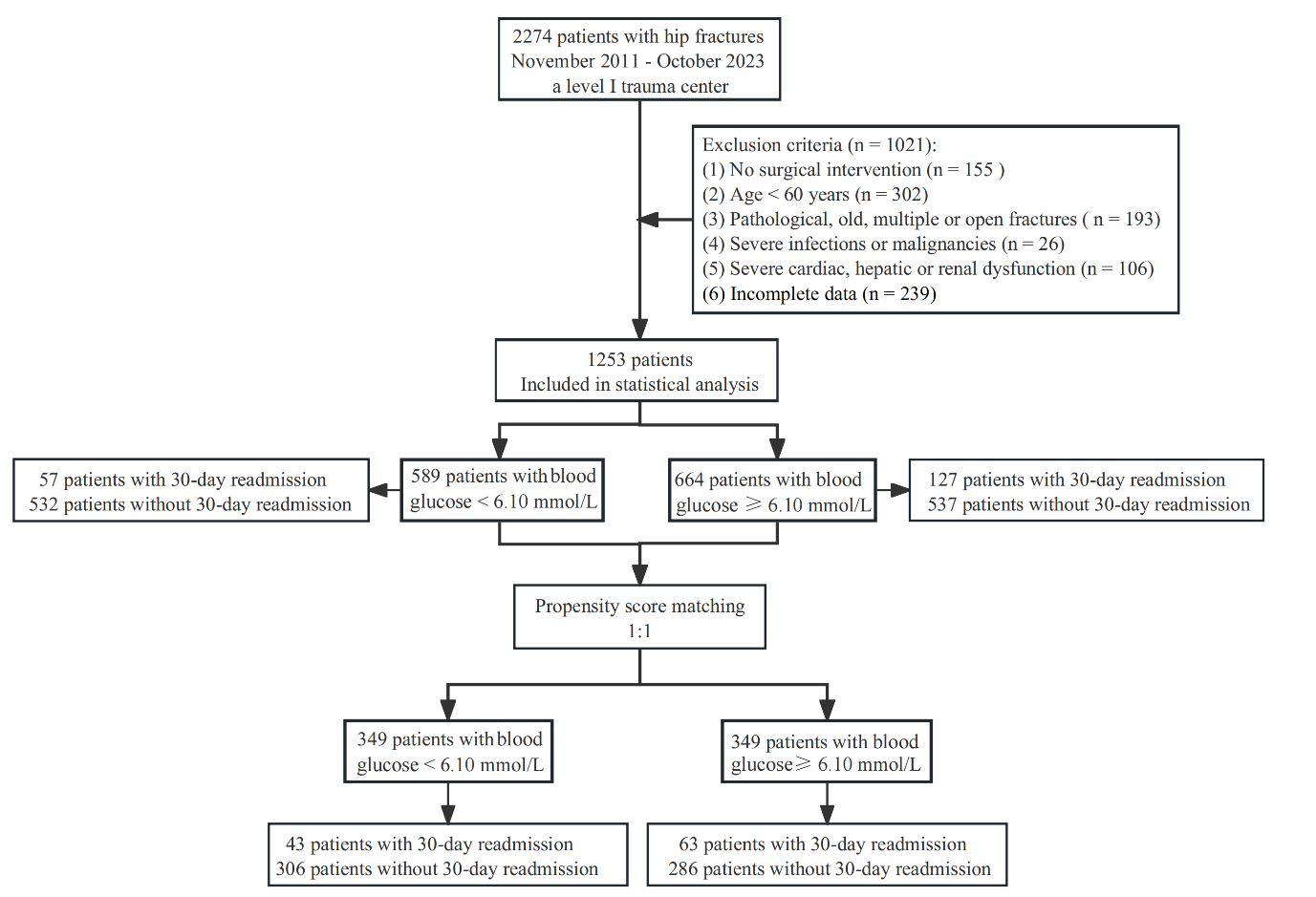
eFigure 1** Flow diagram of enrollment and PSM.

**eFigure 2** The prediction model of admission blood glucose levels for 30-day readmission rate after hip fracture.

**
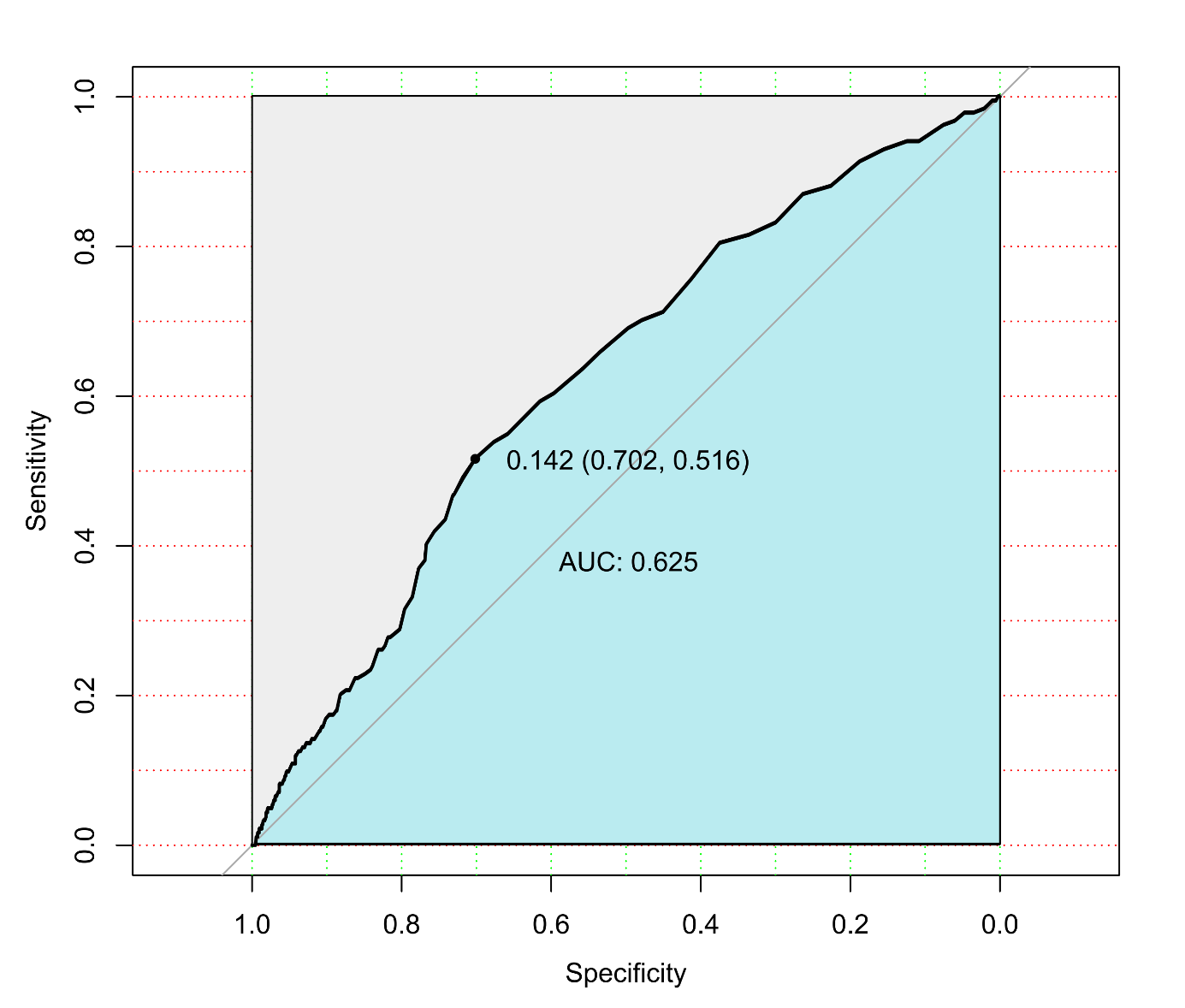
**

**eTable 1 Baseline characteristics of the patients between 30-day readmission and non-readmission groups**

| Variables | Total patients  (n = 1253) | Groups | | ^†^P for Trend |
| --- | --- | --- | --- | --- |
|  |  | Without readmission  (n = 1069) | 30-day readmission  (n = 184) |  |
| Demographic |  |  |  |  |
| Male gender (n, %) | 499 (39.8) | 436 (40.8) | 63 (34.2) | 0.094 |
| Age, × year (Mean, SD) | 74.72 (9.58) | 73.97 (7.54) | 79.10 (8.66) | <0.001 |
| Smoking (n, %) | 215 (17.2) | 187 (17.5) | 28 (15.2) | 0.450 |
| Alcohol (n, %) | 146 (11.7) | 129 (12.1) | 17 (9.2) | 0.269 |
| Comorbidities |  |  |  |  |
| Hypertension (n, %) | 632 (50.4) | 515 (48.2) | 117 (63.6) | <0.001 |
| Diabetes (n, %) | 286 (22.8) | 230 (21.5) | 56 (30.4) | 0.008 |
| COPD (n, %) | 146 (11.7) | 118 (11.0) | 28 (16.2) | 0.103 |
| Cardiovascular disease (n, %) | 384 (30.6) | 316 (29.6) | 68 (37.0) | 0.044 |
| Stroke (n, %) | 328 (26.2) | 258 (24.1) | 70 (38.0) | <0.001 |
| Dementia(n, %) | 48 (3.8) | 37 (3.5) | 11 (6.0) | 0.100 |
| Intracerebral hemorrhage (n, %) | 67 (5.3) | 49 (4.6) | 18 (9.8) | 0.004 |
| Chronic liver disease (n, %) | 58 (4.6) | 47 (4.4) | 11 (6.0) | 0.346 |
| Chronic kidney disease (n, %) | 64 (5.1) | 48 (4.5) | 16 (8.7) | 0.017 |
| Operation |  |  |  |  |
| Fracture type |  |  |  |  |
| Femoral neck fracture (n, %) | 663 (52.9) | 559 (52.3) | 104 (56.5) | 0.537 |
| Intertrochanteric fracture (n, %) | 516 (41.2) | 447 (41.8) | 69 (37.5) |  |
| Subtrochanteric fracture (n, %) | 74 (5.9) | 63 (5.9) | 11 (6.0) |  |
| Surgery type |  |  |  |  |
| Total Hip Arthroplasty (n, %) | 160 (12.8) | 138 (12.9) | 22 (12.0) | <0.001 |
| Hemiarthroplasty (n, %) | 309 (24.7) | 240 (22.5) | 69 (37.5) |  |
| Intramedullary nail fixation (n, %) | 414 (33.0) | 360 (33.7) | 54 (39.3) |  |
| Internal fixation with steel plate (n, %) | 166 (13.2) | 142 (13.3) | 24 (13) |  |
| Internal fixation with hollow nails (n, %) | 204 (16.3) | 189 (17.7) | 15 (8.2) |  |
| Intraoperative blood loss, ×ml (Mean, SD) | 175.20 (153.37) | 173.62 (152.81) | 184.40 (156.74) | 0.379 |
| Transfusion (n, %) | 208 (16.6) | 171 (16.0) | 37 (20.1) | 0.166 |
| Postoperative ICU (n, %) | 59 (4.7) | 50 (4.7) | 9 (4.9) | 0.899 |
| Admission time |  |  |  |  |
| ＜6 hours (n, %) | 666 (53.2) | 574 (53.7) | 92 (50.0) | 0.051 |
| 6-24 hours (n, %) | 192 (15.3) | 171 (16.0) | 21 (11.4) |  |
| ≥ 24 hours (n, %) | 395 (31.5) | 324 (30.3) | 71 (38.6) |  |
| Bedridden time, ×day (Mean, SD) | 5.90 (4.02) | 5.86 (4.07) | 6.11 (3.75) | 0.437 |
| Intraoperative time, ×hour (Mean, SD) | 1.66 (0.80) | 1.68 (0.82) | 1.57 (0.71) | 0.091 |
| ASA classification |  |  |  |  |
| Ⅲ-Ⅳ (n, %) | 705 (56.3) | 562 (52.6) | 143 (77.7) | <0.001 |
| Ⅰ-Ⅱ (n, %) | 548 (43.7) | 507 (47.4) | 41 (22.3) |  |
| Laboratory findings |  |  |  |  |
| WBC count, ×10^9/L (Mean, SD) | 8.86 (2.86) | 8.80 (2.86) | 9.20 (2.90) | 0.082 |
| HGB level, ×g/L (Mean, SD) | 119.72 (20.58) | 120.54 (20.44) | 114.92 (20.80) | 0.001 |
| Albumin, ×g/L (Mean, SD) | 37.95 (4.62) | 38.20 (4.55) | 36.49 (4.76) | <0.001 |
| BUN, ×mmol/L (Mean, SD) | 7.40 (4.87) | 7.32 (4.85) | 7.87 (5.00) | 0.154 |
| Cr, ×umol/L (Mean, SD) | 72.37 (64.79) | 72.59 (68.77) | 71.04 (33.52) | 0.764 |
| D-Dimer, ×mg/L (Mean, SD) | 4.94 (5.04) | 4.88 (5.02) | 5.31 (5.18) | 0.281 |
| Common complication |  |  |  |  |
| DVT (n, %) | 160 (12.8) | 126 (11.8) | 34 (18.5) | 0.012 |
| UTI (n, %) | 289 (23.1) | 237 (22.2) | 52 (28.3) | 0.070 |
| Pneumonia (n, %) | 114 (9.1) | 82 (7.7) | 32 (17.4) | <0.001 |

^†^P values are from T-test for continuous variables and from the chi-square test for categorical variables.

Abbreviations: SD, Standard deviation; COPD, Chronic obstructive pulmonary disease; ASA: the American Society of Anesthesiologists Physical Status Classification System; WBC, White blood cell; HGB, hemoglobin; BUN, Blood urea nitrogen; Cr, Creatinine; DVT, Deep Vein Thrombosis; UTI, Urinary Tract Infection
